# Financial Literacy and Gambling Behaviour: A Systematic Review

**DOI:** 10.1007/s10899-025-10426-7

**Published:** 2025-09-29

**Authors:** Fiona O’Connor, Nicola Ivory

**Affiliations:** Charles Sturt University, Bathurst, NSW Australia

**Keywords:** Gambling, Financial literacy, Financial knowledge, Systematic review

## Abstract

Low financial literacy may contribute to poor financial decision-making and has been investigated as a potential risk factor for problematic gambling. This systematic review examined the association between financial literacy and gambling. PsycInfo, Academic Search Complete, CINAHL, Psychology and Behavioural Sciences Collection, SocIndex, Web of Science Core Collection, Scopus, and Proquest Psychology databases were searched for peer-reviewed empirical studies published in English, reporting on the association between financial literacy and gambling frequency and/or problems. 115 studies were retrieved with nine meeting the inclusion criteria. Most studies measured objective financial knowledge (e.g., Big 3 or Big 5), however a few measured subjective confidence in financial matters or both. Sample sizes ranged from a few hundred to tens of thousands. Most studies assessed frequency of gambling as their gambling outcome. Only one study employed a comprehensive measure of problematic gambling behaviour. Financial literacy was generally negatively associated with gambling, although one study reported a positive association. Findings of this review suggest increased financial literacy may be associated with reduced gambling frequency, highlighting potential for financial literacy education interventions to reduce gambling-related harms. However, this conclusion derives from only a handful of cross-sectional studies that are at times limited by incomplete or imprecise reporting, the use of brief financial literacy measures, and a lack of focus on gambling problems in addition to gambling frequency. Further research overcoming these limitations is needed to strengthen confidence in these conclusions.

**Supplementary Information:**

The online version contains supplementary material available at 10.1007/s10899-025-10426-7.

## Introduction

Gambling is a popular recreational activity for many the world over. While most do so without experiencing problems, for others, gambling can turn into a problematic behaviour with serious consequences. Gambling behaviour becomes problematic when it is characterised by persistent and compulsive gambling that persists despite detrimental effects (Jazaeri & Bin Habil, 2012). Individuals struggling with their gambling behaviour may have a desire to stop but be preoccupied by thoughts and urges to gamble (Jazaeri & Bin Habil, 2012).

Problem Gambling has been defined as “gambling behaviour that creates negative consequences for the gambler, others in his or her social network, or for the community” (Ferris & Wynne, 2001, p. 8). It is a broad umbrella term that encompasses both gambling disorder, defined as “persistent and recurrent problematic gambling behaviour leading to clinically significant impairment or distress” (DSM-5-TR, American Psychiatric Association, 2022), and non-clinical individuals affected by gambling problems (Gainsbury et al., 2014). When gambling behaviour becomes excessive, it can lead to high levels of debt and financial strain (e.g., Marionneau et al., 2023). Higher levels of financial literacy have been shown to be associated with reduced financial stress (Steen & MacKenzie, 2013), and therefore may offer protective benefits against financial strains associated with excessive gambling behaviour.

Gambling behaviour has been shown to be consistently and significantly related to financial motives (e.g., Tabri et al., 2022; Maclaren et al., 2015). A study of self-generated reasons for gambling found that 46% of the respondents identified financial motives, or gambling motivated by the desire to win money, as the primary reason for gambling (McGrath et al., 2010). Given that people with higher financial literacy are more likely to understand that gambling is not a financially profitable activity due to the extremely low odds of winning, a reasonable argument is that increased levels of financial literacy could lower levels of financially motivated gambling.

Financial literacy is generally understood to reflect the “knowledge and understanding of financial concepts and risks, and the skills, motivation and confidence to apply such knowledge and understanding in order to make effective decisions across a range of financial contexts” (OECD, 2014, p. 33). However, how best to measure financial literacy is challenging as there is no widely agreed upon definition (Huston, 2010). Most research on financial literacy adopts an objective approach, seeking to measure people’s financial knowledge or understanding (Allgood & Walstad, 2015). In research adopting this approach, financial Literacy is often measured by three questions, known as the Big 3, designed to target understanding of inflation, compound interest, and diversification respectively (Lusardi & Mitchell, 2007). For example, the first question states “Suppose you had $100 in a savings account and the interest rate was 2% per year. After 5 years, how much do you think you would have in the account if you left the money to grow?” Respondents are required to select the correct answer from a selection of possible answers. The Big 5, an extension of the Big 3, is another popular measure which includes the original three questions plus two additional items; one on bond prices and another on mortgages (Hastings et al., 2013). For both measures, a total financial Literacy score is calculated by summing the number of questions answered correctly. The Big 3 and the Big 5 were designed to be a brief and simple approximation of financial literacy. However, the brevity of these scales may present some issues, as financial literacy can arguably be considered a broad concept that is unlikely to be captured fully by just three or five items. In order to address this, some researchers have developed more detailed measures (e.g., Amonhaemanon, 2022; 2023; Cho, 2022). However, this has created marked variability in how financial literacy has been defined and measured across studies.

One dimension along which approaches to measuring financial literacy vary is the distinction between subjective self-assessments and more objective measures. Subjective financial literacy is a self-report perception or evaluation of one’s own financial skills, while objective measures involve an external assessment or knowledge test of a person’s actual understanding of financial concepts. While subjective perceptions or confidence in financial knowledge and objective measures of financial literacy are likely to be positively correlated, they represent distinct constructs that are unlikely to always align perfectly. For example, it is possible for individuals to exhibit varying combinations of the two, such as high subjective confidence paired with low factual knowledge, or conversely low subjective confidence paired with high factual knowledge.

Consequently, studies have measured financial literacy in different ways, some focusing on subjective perceptions or confidence in financial knowledge (e.g., Allgood & Walstad, 2015), while others relying upon objective tests of actual financial Literacy such as the Big 3 (e.g., Becchetti et al., 2018). An even smaller number of studies have chosen a more indirect approach, measuring financial behaviours presumed to reflect levels of financial literacy, such as paying bills on time or being aware of how much savings one has (e.g., Shin et al., 2015). Even in the more objective approaches, which measure financial literacy knowledge, the number of items can vary from two (Razen et al., 2021), to as many as 20 (Wang et al., 2023), creating variability across studies in terms of the breadth of the construct that is being captured. The lack of an agreed definition and approach to measuring financial literacy has been argued to inhibit the ability to make more meaningful comparisons across studies (Huston, 2010). Particularly when comparing the financial literacy of different countries, where inconsistencies across studies make it difficult to discern what is a meaningful cultural difference and what reflects measurement variability.

Despite these challenges, global survey data suggests generally low levels of financial literacy worldwide (e.g. Klapper et al., 2015; OECD, 2016). For example, using consistent measures, the OECD (2016) reported low financial Literacy among adults across 30 countries. However, financial literacy rates also appear to vary somewhat across countries. For example, Klapper et al. (2015) found relatively low rates of financial literacy in South Asian countries (25% or less), compared to countries Like Australia, Canada, and the United Kingdom, where rates were around 65%.

As the financial world continues to grow in complexity, the importance of financial literacy in navigating daily financial decisions is similarly increasing (Allgood & Walstad, 2015). Indeed, evidence indicates that financial literacy is associated with improved financial wellbeing (Zhang & Chatterjee, 2023). Financially literate individuals are more likely to plan for retirement (Lusardi & Mitchell, 2007) and accumulate more wealth than people lower in financial literacy (Behrman et al., 2012). Low levels of financial literacy have been associated with ineffective spending, poor debt management, and poor financial planning (Lusardi, 2019), which can be costly in both the short and long-term (Allgood & Walstad, 2015). While there is a significant and growing body of evidence to support the importance of the relationship between financial literacy and financial behaviours, research examining the relationship between gambling and financial literacy is still in its infancy.

In comparison to other financial behaviours, the relationship between gambling and financial literacy is likely to be particularly nuanced or complex, as gambling might not always be considered a financial behaviour. That is, while gambling has been linked to financial or winning motivations (Francis et al., 2015; Wardell et al., 2015), and for these individuals, increasing financial literacy may offer protective effects against problem gambling as a financial behaviour. Many individuals gamble for other reasons, such as for social reasons, for entertainment, for distraction from negative emotions, or enhancement of positive emotions, and it is possible that increased financial literacy may not have a protective effect against excessive gambling in these cases, where gambling for these individuals might not be considered a financial behaviour. Therefore, it is important to examine the evidence linking financial literacy to gambling behaviour as well as the interactions between financial literacy and the motivations underlying the gambling behaviour.

Thus, the purpose of the current review is to present the state of research on the relationship between financial literacy and gambling. The research question explored in this review is whether financial literacy offers a protective benefit against problematic gambling. Financial literacy has only relatively recently emerged as a research topic of interest, but is recognised as an increasingly important competency, given the ubiquity of participation in financial decision making in the modern world. Similarly, problem gambling has seen a surge in research since its inclusion as a recognised disorder within the Diagnostic and Statistical Manual of Mental Disorders (DSM-5; American Psychiatric Association, 2013). Therefore, while research addressing both topics are still somewhat in their infancy, it is arguably timely to explore this emerging research now to identify strengths and gaps in the knowledge base, as well as to support standardisation of research methodologies.

## Method

### Search Strategy and Selection Criteria

A systematic search was conducted in January 2024 to identify peer-reviewed, empirical papers published in English that examined the association between financial Literacy and gambling behaviour or problem gambling. PsycInfo, Academic Search Complete, CINAHL, Psychology and Behavioural Sciences Collection, SocIndex, Web of Science Core Collection, Scopus and Proquest Psychology databases were searched. All searches were conducted on 17 January 2024, searching for literature from the commencement of each database. Reference lists of all included studies, as well as any review papers found in the search, were harvested for additional eligible studies.

A review protocol was developed which detailed the databases to be searched and an exhaustive set of search terms relating to two themes: (1) financial literacy and (2) gambling behaviours or problems. This search was entered into all databases, as shown below:

(“financial literacy” OR “financial wellness” OR “financial wellbeing” OR “financial knowledge”) AND (gambling OR PGSI OR IGT OR SOGS)

This review was conducted in accordance with the Preferred Reporting Items for Systematic Reviews and Meta-Analyses (PRISMA) statement. Covidence, an online collaboration software platform designed for use in systematic reviews, was used to conduct the screening process. All abstracts were screened independently by both authors. Following abstract screening, full-text review of the remaining articles was done to determine the final list of included studies. Discrepancies were identified and resolved by mutual assessment against an agreed protocol between the authors. The abstract and full-text screening processes were evaluated for inter-rater reliability using Cohen’s Kappa, yielding values of 0.68 and 0.60 respectively. These statistics fall within the “moderate” range and are considered acceptable (McHugh, 2012). Studies were excluded if they were not published in English, were not peer reviewed, and did not include a measure of financial literacy or either gambling frequency or gambling problems.

### Data Extraction and Synthesis

Data extracted from included studies encompassed author name(s), date of publication, country, study aim, sample size, participant characteristics (e.g., age, sex), financial literacy measure, gambling/gambling problems measure, and results regarding the association between financial literacy and gambling. The methodological strengths and weaknesses of the included studies were qualitatively evaluated.

## Results

**Fig. 1:**
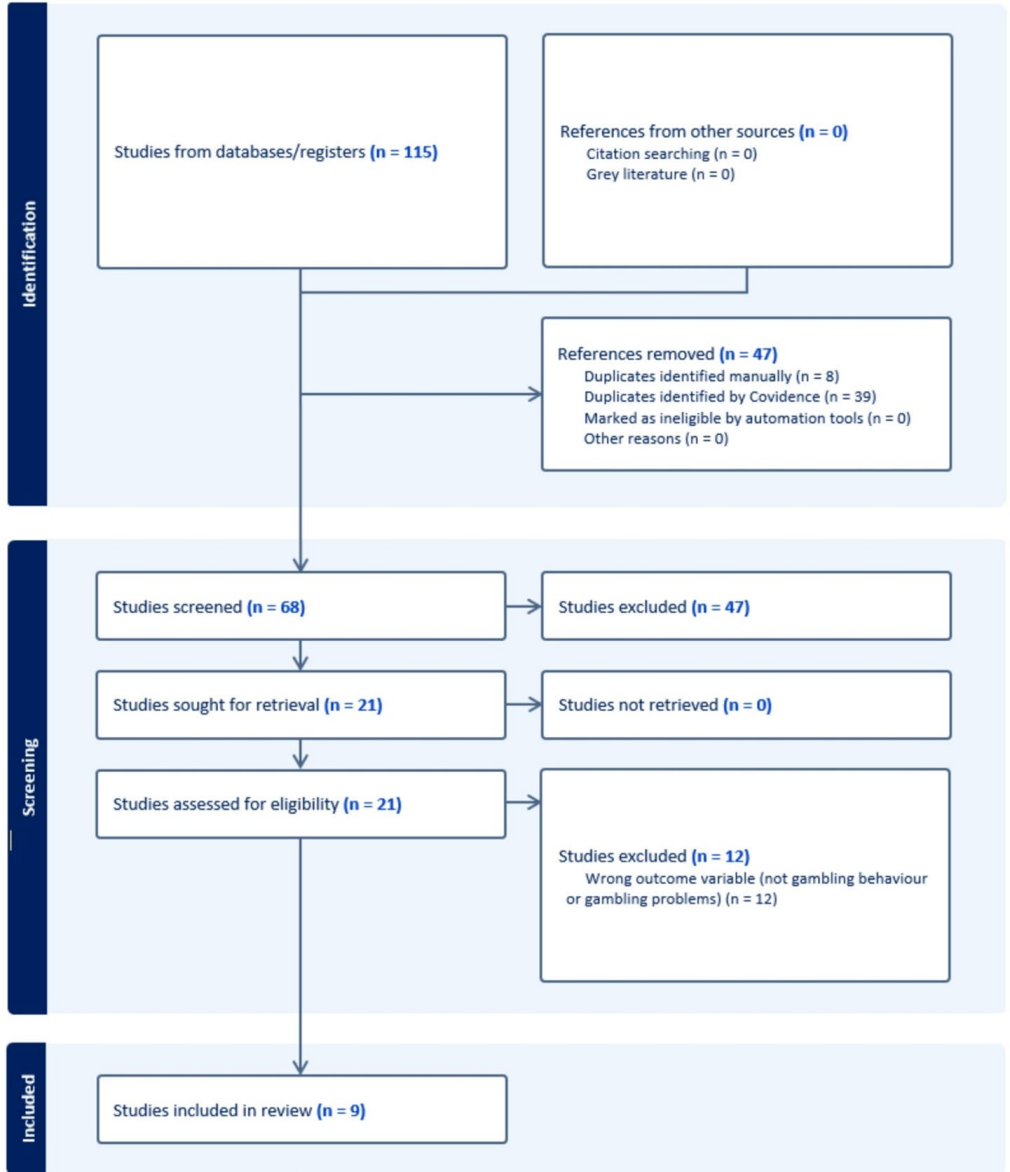
PRISMA statement flow diagram: summary of systematic search and review process

The search identified 115 papers. Seventy-six papers remained for abstract and title screening following removal of duplicates. Forty-seven papers were excluded following title and abstract screening and a further 12 removed following full text review, resulting in 9 articles retained as meeting the inclusion criteria (see Fig. 1). It should be noted that two studies published by Amonhaemanon may have been based on the same dataset as the sample sizes are identical, although we could not find any commentary to that effect in either publication. As such, these studies will be discussed as if they have been conducted on independent samples. Year of publication ranged from 2018 to 2023, with the majority of articles (89%) published since 2021. Of the nine articles reviewed, three were conducted in the United States of America, two in Thailand, and one each in Austria, China, Italy, and Japan. Sample sizes ranged from 627 to 26,566. Six studies reported the mean age and standard deviation of the sample and a further two reported percentages within age ranges. One study provided no information regarding age of the sample. Most of the studies recruited adults; with the sample mean ages ranging from the mid to late 40s. One study was conducted on adolescents. Information relating to sex/gender was not reported in all papers but was able to be sourced for all studies from online supplementary materials where necessary. The proportion of males in the samples ranged from 27 to 83%. Further sample characteristics of the included studies are summarised in Table 1.

**Table 1:** Overview of included studies

	Author	Year	Key findings	*N*	M_age_(SD)	Sex (male)	Country
*Studies of gambling frequency*							
1	Amonhaemanon	2022	Self-assessed subjective confidence in financial literacy (scores range from 0 = no Literacy to 10= high literacy) negatively predicted odds of being an at-risk gambler (defined as buying the lottery frequently but not at every draw). Objective financial literacy knowledge (assessing 7 topics - the Deposit Protection Agency (DPA) and the Credit Bureau, compound interest, inflation, purchasing power, risk and return from investment, and risk diversification) was not a significant predictor of being an at-risk gambler.	995	nr	27%	Thailand
2	Amonhaemanon	2023	Objective financial literacy knowledge (10 items assessing 7 topics - the Deposit Protection Agency (DPA) and the Credit Bureau, compound interest, inflation, purchasing power, risk and return from investment, and risk diversification. Converted into a categorical variable; respondents scoring above the mean categorised as high literacy and those below the mean as low literacy) was negatively associated with gambling intensity (operationalised as the product between frequency of gambling and amount of money spent gambling). A significant chi square revealed people low in financial literacy knowledge were more likely to have high gambling intensity (38%) than low gambling intensity (30%). While people high in financial literacy knowledge were more likely to have low gambling intensity (33%) versus high gambling intensity (27%).	995	15–22: 47% 23–39: 17% 40–54: 19% 55–73: 17%	27%	Thailand
3	Becchetti et al.	2018	Financial literacy (Big 3) was negatively associated with the frequency of two different modes of gambling. A chi square revealed that scratch-off players were significantly less Likely to answer all three questions on the big 3 correctly than non-gamblers (27% versus 37%, χ^2^ = 4.56, *p* <.05). Slot/video poker players were also significantly less Likely to answer all 3 questions correctly than non-gamblers (24% versus 37%; χ^2^ = 5.40, *p* <.05).	423^	35.62(12.65)	47%	Italy
4	Cho	2022	An ordered probit model with an endogenous regressor (average education level of a zip code area) revealed an increase in financial literacy was associated with decreased frequency of lottery consumption. For each lottery consumption frequency (never, less than a month, about once a month, about once a week, a few times a week, almost every day), an increase in financial literacy decreased the frequency of lottery consumption. However, higher financial literacy had a stronger effect on reducing less frequent lottery consumption. That is, the effect of increased financial literacy decreasing gambling behaviour weakened as the frequency of gambling increased.	26,566	18–24: 12% 25–34: 18% 35–44: 16% 45–54: 17% 55–64: 18% *≥* 65: 19%	49%	USA
5	Razen et al.	2021	Financial literacy (number of financial literacy questions correctly answered) was a positive, but not statistically significant, predictor of frequency of gambling in an adolescent sample using ordered logistic regression. However, they did find self-report risk-taking in financial matters was positively and significantly associated with gambling.	627	15.8(1.9)	40%	Austria
6	Watanapongvanich et al.	2021	Financial literacy (Big 3) was a negative but not significant predictor of gambling frequency. Father’s education, as an instrumental variable to address potential endogeneity, was found to significantly negatively predict gambling frequency at the *p* <.001 level. Financial education was found to have no significant association with gambling frequency.	3,687	49.82(12.64)	49%	Japan
7	Watanapongvanich et al.	2022	Financial Literacy, when measured using the Big 3, was a negative but not significant predictor of gambling frequency in both states with and without EGMs. The association between financial literacy when measured as financial education level and gambling frequency was also not statistically significant in states with or without EGMs (*p* <.10*).	4,215	49.54(15.91)	46%	USA
*Studies of gambling problems*							
8	Grable et al.	2021	Gambling affinity (i.e., how likely is it that you would bet a day’s income?) was positively correlated with subjective perceptions of financial knowledge (i.e., how knowledgeable are you about finance topics). In a regression, financial literacy continued to positively predict gambling affinity after controlling for a wide range of known demographics.	>500^^	45.08(16.53)	49%	USA
9	Wang et al.	2023	t-tests showed lottery consumers (people who gambled at any level) scored significantly lower on financial knowledge, financial capacity, financial management values, wealth values and financial ethics than the general population (medium effect size). Two of the five dimensions of financial literacy (ethics and wealth values) significantly negatively correlated with problem gambling, measured using the 19-item Scale of Assessing Problem Gambling (SAPG; Li et al., 2012). The other three dimensions of financial literacy (knowledge, capacity, management values) were negatively correlated with problem gambling but not significant.	10,374 (*n* = 316 gamblers)	40.43(10.95)	83%	China

Note: nr = not reported. ^ = sample size not reported in the paper - this value was obtained from the data provided as Supplementary material to the paper online; ^^ = sample size imprecisely reported in the article as “slightly more than 500 adults”; * = The authors of the original study report the results of this analysis as being statistically significant at the p less than 0.10 level. This is not a widely accepted cut-off for statistical significance; therefore, the current authors have described the correlation as not statistically significant according to standard convention of a cut-off of 0.05. It should also be noted there was some inconsistency in reporting of this finding in the original paper - stating in different sections that the correlation was only significant in states with EGMs and then also contradictorily, that it was only significant in states without EGMs. The authors were contacted for clarification however no response was received. As the level of statistical significance, the authors used to determine significance is higher than that accepted by the current authors, this inconsistency does not affect the conclusions we draw regarding the findings, although it does affect the conclusions that the original authors drew

### Key Findings: Associations between Financial Literacy and Gambling

While the research findings were somewhat mixed, the majority of studies found increased financial literacy was negatively and significantly associated with gambling behaviour (*n* = 6; Amonhaemanon, 2022; 2023; Becchetti et al., 2018; Cho, 2022; Wang et al., 2023; Watanapongvanich et al., 2021). Two of these studies found this negative association between financial literacy and gambling when using education level as an instrumental variable to address potential endogeneity between financial literacy and gambling behaviour (i.e., Cho, 2022; Watanapongvanich et al., 2021). An additional two studies found a negative relationship between financial literacy and gambling which did not reach statistical significance (Razen et al., 2021; Watanapongvanich et al., 2022), while only one of the nine studies found a positive and statistically significant relationship between financial literacy and gambling behaviour (Grable et al., 2021). No studies found a positive but not significant association between financial literacy and gambling.

### Measurement of Financial Literacy

The majority of studies (*n* = 8) measured actual or objective financial literacy with items that test knowledge of financial literacy concepts (i.e., Amonhaemanon, 2022; 2023; Becchetti et al., 2018; Cho, 2022; Razen et al., 2021; Watanapongvanich et al., 2021; 2022; Wang et al., 2023). All but one of these studies (Wang et al., 2023) drew upon Lusardi and Mitchell’s research, using either the Big 3, the Big 5, or some combination therein, sometimes adding items to these widely recognised scales. Specifically, three studies employed the Big 3 (Becchetti et al., 2018; Watanapongvanich et al., 2021; 2022), two studies administered the Big 3 but also included additional items (Amonhaemanon, 2022; 2023), one study used only two of the three items from the Big 3 (Razen et al., 2021), and one study utilised the Big 5 with the addition of one extra item (Cho, 2022). Wang et al. (2023) employed a comprehensive multidimensional measure of financial literacy, which included five factors: Financial Knowledge (20 items), Financial Capacity (15 items), Financial Management Values (12 items), Financial Ethics (9 items), and Wealth Values (9 items). Two studies measured self-report perceptions of financial literacy or financial literacy confidence (Amonhaemanon, 2022; Grable et al., 2021). These studies used a single item that asked participants to report their perceived financial literacy (e.g., “How knowledgeable are you about personal finance topics?” Grable et al., 2021).

### Measurement of Gambling Behaviour

The majority of studies (*n* = 7) measured gambling behaviour in terms of frequency. Several of these studies only asked a single question to measure gambling frequency (i.e., Watanapongvanich et al., 2022; Razen et al. 2021; Cho, 2022) and these questions varied across studies. For example, Cho (2022) employed an objective time frequency (i.e., daily, weekly, monthly etc.), while Amonhaemanon (2022; 2023) considered the frequency of participation in the national lottery draw (i.e. every draw, most draws etc.). Amonhaemanon (2023) also measured the amount of money spent gambling within the past year and multiplied this by the frequency of gambling to create a composite variable which they called gambling intensity. Razen et al. (2021) measured frequency subjectively with a Likert scale (i.e., never, seldom, occasionally, etc.).

Only two studies attempted to measure whether the gambling was problematic (Wang et al., 2023; Grable et al., 2021). Wang et al. (2023) administered a 19-item scale to measure four dimensions of gambling behaviour: (1) impact on life, work, and relationships; (2) excessive win expectations (whether players expected to win excessively); (3) compulsive disorder (measuring difficulty controlling gambling); and (4) depressive signs (measuring negative emotions regarding gambling). Grable et al.’s (2021) approach was less direct but could be argued to indirectly tap into problem gambling. They used a single item asking participants how likely they would gamble a day’s wages at the casino (from 1 = very unlikely to 10 = very likely). Gambling such a large amount of money in a very short period of time could be considered a risky behaviour that is likely to be positively associated with problem gambling.

### Methodological Quality

The methodological quality of the included studies was generally very good. Key strengths included the use of very large sample sizes and strategies to control for potential confounding variables in most studies, such as including an endogenous regressor within a probit model. However, reporting of sample characteristics was often unclear or incomplete, with many studies omitting key demographic information. While this doesn’t necessarily reduce the quality of the research itself, it does make it a little harder to properly interpret the findings or assess their generalisability to other populations.

Only one study provided information regarding the reliability or validity of their measures (i.e., Wang et al., 2023). While internal consistency was rarely reported, this largely reflects the use of very short scales, often single items, to measure financial literacy and/or gambling behaviour. However, greater attention to psychometric reporting, particularly validity, is still warranted. Future research in this area would benefit from the use of larger, multi-item scales that better support the evaluation and reporting of reliability and validity.

## Discussion

To the authors knowledge, this is the first systematic review to summarise the body of literature examining the association between financial literacy and gambling behaviour. The results of this review suggest that higher levels of financial literacy may be associated with lower frequency of gambling and to a much lesser extent, lower likelihood of gambling-related problems. However, these conclusions are based on a very limited number of studies, most of which did not measure gambling problems per se. Thus, we recommend that these conclusions and interpretations are accepted with some caution.

All but one of the studies found a negative relationship between financial literacy and gambling, with the findings suggesting that gamblers tend to have lower levels of financial literacy on average compared to non-gamblers (Becchetti et al., 2018; Wang et al., 2023) or that higher levels of financial literacy are associated with lower frequency of gambling (e.g., Cho, 2022; Watanapongvanich et al., 2021). This is consistent with research that suggests increased financial literacy has a positive effect on financial decision-making and behaviours (e.g., Lusardi, 2019; Lusardi & Mitchell, 2009; Lusardi & Tufano, 2015). However, one study in this review identified financial literacy as a positive predictor of problem gambling (Grable et al., 2021). Specifically, they found subjective perceptions of financial knowledge positively predicted the likelihood of engaging in a risky gambling behaviour, that is, betting a day’s income at the casino. Notably, only one other study in this review measured problem gambling, as opposed to gambling frequency (Wang et al., 2023), however that study found negative associations between financial literacy and problem gambling, consistent with the majority of other studies in this review. The limited number of studies focusing on problem gambling rather than gambling frequency challenges the robustness of the evidence for financial literacy as a protective factor against problematic gambling. Most of the existing research has centered on gambling frequency, which may not fully capture the severity or harms associated with gambling behaviours. Consequently, while financial literacy appears to show protective effects against gambling frequency, its role in mitigating problem gambling remains less well understood.

There are several possible explanations for the positive association found between financial literacy and problem gambling by Grable et al. (2021). Firstly, financial literacy may be negatively associated with gambling frequency but positively associated with problematic gambling. While these findings may seem contradictory, they could suggest that the association between financial literacy and gambling behaviour varies based on the severity of the gambling behaviour. For example, once gambling reaches problematic or pathological levels, rational thought (i.e. greater financial literacy) may no longer be helpful in reducing it, an argument previously expressed by others (Watanapongvanich et al., 2022; Cho, 2022). Secondly, high levels of financial literacy may lead to overconfidence in financial decision-making capacity which could impair decision-making in a way that leads to taking greater financial risks in people who gamble at problematic or pathological levels. Thirdly, subjective evaluations of financial knowledge may not correlate well with objective measures of financial literacy. That is, while high confidence in financial literacy was found to positively correlate with problem gambling, the actual or objective financial literacy of these participants was not measured and is not known. Their subjective evaluations may have represented an inaccurate or overestimate of their actual financial literacy levels. While these possibilities may explain this discrepant finding, given this discussion is based around a single study that found contradictory results, it is important not to place too much weight on this single finding. It is also possible that the positive association is simply an outlier result. Replication studies are needed to better understand how financial literacy may be associated with problem gambling versus frequency of gambling. Future research should strive to include both frequency-based and problem-focused measures to provide a more comprehensive understanding of the potential protective role of financial literacy.

Cultural differences across the studies may also help explain the current findings. Research shows there are cultural differences in rates of gambling (e.g., Raylu & Oei, 2004) and there is evidence that the gambling patterns of populations are shaped by cultural and historical contexts as well as variations in regulatory policies between countries (e.g., Kairouz et al., 2016). As such, it is possible that the relationship between financial literacy and gambling may be different in countries where gambling is more readily accepted, widely available, and legal, such as the United States and Australia, versus countries which have stricter laws and/or largely prohibit gambling, such as China. For example, increasing financial literacy in cultures with generally positive attitudes towards gambling and easy accessibility to gambling venues may not reduce frequency of gambling. Due to wide social acceptance of gambling in these cultures, motivations for gambling may often be for entertainment reasons rather than financial reasons. In contrast, increasing financial literacy may offer greater protective effects against gambling behaviour in countries where gambling is more widely perceived as a negative financial behaviour and is less socially accepted. Further research is needed which explores the interplay among social attitudes towards the acceptability of gambling, availability of gambling, motives for gambling and financial literacy, to further explicate these ideas. While the studies included in this review originate from five different countries, there are too few studies in the current review to engage in any meaningful discussion of the role that culture may play in the associations between financial literacy and gambling behaviours, however the potential impact of cultural differences in gambling behaviours is important to be acknowledged and explored in future studies.

Cultural and regulatory differences also appear to influence how various forms of gambling, such as lottery participation, are perceived and studied across countries. We noted that several studies considered lottery purchases as their sole form of gambling behaviour (e.g., Amonhaemanon , 2022, 2023; Cho 2022). This finding stood out to the current researchers, as lottery ticket purchasing in Australia, where the authors are located, is typically regarded as a low-risk form of gambling (Armstrong & Caroll, 2017). In Australia, lottery gambling is rarely associated with problems and is seldom the primary focus of research given the ubiquity of the behaviour and its non-significant relationship with problem gambling (Browne et al., 2023). Lottery gambling is considered a low-risk form of gambling primarily because it lacks a continuous-play format. Continuous-play gambling, such as Electronic Gaming Machines (EGMs) or pokie machines, is characterised by a high play-rate and minimal delay between the bet and outcome. This gambling format has been shown to affect brain structures in ways that perpetuate compulsive gambling (Murch & Clark, 2016).

The delayed outcome of a lottery ticket purchase is unlikely to perpetuate compulsive gambling in this way. Thus, it is possible that by only capturing lottery purchases and not other forms of gambling, a number of the studies included in this review may not be capturing the most problematic forms of gambling. Notably, cultural differences might mean that lottery ticket purchases have stronger associations with gambling problems in some countries. However, given the delay of a lottery ticket outcome versus continuous play format games, it is unlikely that the comparison between these two gambling behaviours could be considered completely equal. Increasing financial literacy could be more likely to impact a pre-meditated behaviour such as lottery ticket purchases (Amonhaemanon, 2022, 2023; Cho 2022), than a compulsive and inherently addictive behaviour such as EGM use or online gambling (Grable et al., 2021; Watanapongvanich et al., 2021). These are interesting questions for future research to explore.

### Limitations

A potential limitation of the current research is the lack of demographic information, some studies did not explicitly report their participant’s basic demographics such as age or sex (e.g., Amonhaemanon, 2022, 2023; Becchetti et al., 2018). The lack of demographic information may negatively affect replicability of the findings. Notably, survey research demonstrates significant differences between men and women’s financial Literacy levels, with 63% of men and 48% of women demonstrating an understanding of the three basic financial literacy concepts (Preston, 2020). Similarly, age may also impact financial literacy, with younger and older people having the lowest levels of financial literacy, with the highest levels occurring during mid-life (Preston, 2020). Younger people have also been shown to have higher rates of problem gambling than older adults (Australian Institute of Health and Welfare, 2023). Considering these known demographic differences, understanding the sample characteristics of the included studies has important implications. However, it should be noted that many of the samples of the included studies were very large which reduces concerns about generalisability.

Unclear or incomplete reporting was a common theme throughout the included studies. For example, it was not always clearly reported how many items were used to measure financial literacy within the studies. As an example, Amonhaemanon (2022) reported that they adapted their measure of financial literacy on the basis of previous work (specifically Lusardi, 2012; Lusardi & Mitchell, 2007; and Bank of Thailand, 2016 as cited in Amonhaemanon, 2022). The exact number of items in the final measure was never clearly reported, with the authors only stating that their measure of financial literacy “addressed seven topics” (Amonhaemanon, 2022, p. 637). This may mean they employed seven items, however the exact number of items nor the possible range of scores on this variable were not reported. This made it challenging to identify if financial literacy in their sample was high or low and how levels of financial literacy in their sample compared to other studies. The heterogeneity in measurement across studies, combined with opaque reporting at times, made drawing comparisons across the studies challenging. This body of literature as a whole would benefit from clearer, more complete, and more transparent reporting of methods.

Additionally, the way in which gambling frequency was defined in some studies could have been stronger. For example, Watanapongvanich et al. (2021) coded their continuous gambling frequency variable into a binary variable: non problem and problem gamblers. Their non-problem gambling group included participants who did not gamble at all with those who gambled once a month or so (and those in between). This grouping is likely too broad, and may have confounded their results, given people who abstain from gambling have been found to be a distinct group from non-problem gamblers in previous research (Ferris & Wynne, 2001).

Of the nine studies, only two specifically sought to capture whether the gambling was problematic (Wang et al., 2023; Grable et al., 2021), with the other studies instead observing gambling frequency. This limits the generalisability of the findings to problem gambling, given gambling frequency and gambling problems are arguably distinct constructs which may relate differently to financial literacy. Given individuals’ varying budgets, differing spend per play, and emotional states or outcomes when gambling, it is reasonable to argue that gambling frequency alone does not adequately capture problematic gambling behaviour. Furthermore, many of the included studies used only a single item to measure gambling frequency, which may not be comprehensive. Thus, there is a need for research in this area to employ measures with a greater range of questions to better capture the nuanced aspects of gambling behaviour and in particular, gambling problems. The Problem Gambling Severity Index (PGSI; Ferris & Wynne, 2001) and South Oaks Gambling Screen (Lesieur & Blume, 1987) are both reliable and valid tools that are used widely in psychological research and would serve as effective and consistent measures for advancing research exploring the association between financial literacy and gambling behaviours. The use of clinician diagnosis for gambling disorder, in addition to these self-report measures, may also be ideal when research budgets permit.

### Areas for Future Research

Further study could explore how financial literacy and gambling motives may interact in the prediction of problem gambling. Independent research has shown financial motives are positively associated with gambling frequency and levels of gambling problems (e.g., see Tabri et al., 2022 for a meta-analysis). Lower levels of financial literacy may be associated with greater endorsement of financial motives for gambling, which may then explain the pathway between low financial literacy and increased gambling. Financial stress may also interact with financial motives and financial literacy to explain gambling behaviours.

Another important consideration for future research is how to measure the construct of financial literacy. While most of the included studies based their questions on Lusardi and Mitchell’s ‘Big 3’ or ‘Big 5’ (Lusardi, 2019), Grable et al. (2021) used a subjective measure to ask participants how financially literate they believed they were. Participants’ confidence in their financial literacy compared to their financial understanding has been shown to differ (Kiliyanni & Sivaraman, 2016), and thus it is important that research seeks to understand both objective and subjective financial literacy and their relationship with gambling behaviour. For example, it is likely that someone with low financial literacy but high confidence in their financial abilities may be most at risk of problem gambling, as they may be less likely to accept advice, seek help for their behaviour, or be receptive to education efforts. While the ‘Big 3’ have been found to be a consistent and useful measure for understanding financial literacy, it is also unlikely that such a short measure is able to accurately capture this broad construct. From a psychometric perspective, a factor with three items may not be particularly stable. A minimum of five items per factor has been recommended (Costello et al., 2005). Given the three questions are argued to measure three unique facets of financial literacy, a stronger scale psychometrically would include at least five items per facet. Therefore, it is promising to see Wang et al. (2023) utilise a more in-depth measure of the financial literacy construct. It may be beneficial for future research to employ this scale in different populations beyond China to further explore utility across diverse samples and cultural contexts.

### Practical Applications

If increased financial literacy does negatively correlate with problematic gambling behaviour, this could guide intervention efforts by focusing on financial literacy education. Indeed, the positive impacts of increased financial literacy in reducing gambling behaviour were demonstrated in a South African study that embedded financial lessons into a nationally televised soap opera (Berg & Zia, 2017). Berg and Zia (2017) found that participants who watched the soap opera with embedded financial literacy content demonstrated improvement in their financial behaviours, including reductions in household gambling. They also found viewers scored significantly higher on financial knowledge following viewing the show. Novel research such as this demonstrates that there may be benefits of financial literacy education as an intervention for problem gambling at a population level.

## Conclusion

Problem gambling is a significant public health concern. This systematic review provides an initial exploration of the association between financial literacy and gambling behaviour. The existing research evidence reviewed here largely shows higher levels of financial literacy may be associated with reduced engagement in gambling, potentially suggesting that improving financial literacy could be an effective strategy to reduce gambling-related problems. However, the current literature is limited in a number of ways related to methodological approach, reporting, and the small number of studies. Given these caveats, the conclusions of this review should be taken tentatively. By synthesising the findings from this literature, this review has provided a foundation and recommendations for conducting, interpreting, and comparing future research in this area. It is recommended that future research extend upon these results to more explicitly explore associations between gambling problems and financial literacy (in addition to frequency of gambling). It is also recommended that research seek to explore the different facets of financial literacy, including detailed measures of both subjective perceptions of financial literacy as well as objective measures of financial knowledge. Future research should employ robust, standardised measures and explore cultural and contextual factors to deepen understanding of this relationship. If financial literacy can be established as a strong predictor of problem gambling, financial literacy education could prove a promising intervention for reducing gambling harms.

## Supplementary Information

Below is the link to the electronic supplementary material.

Supplementary Material 1 (XLSX 35.4 KB)

## Declarations

No human participants were directly involved in the research process.
